# Intraoperative marking of pulmonary nodules in a hybrid operating room: electromagnetic navigation bronchoscopy versus percutaneous marking

**DOI:** 10.3389/fsurg.2024.1482120

**Published:** 2024-09-30

**Authors:** María Teresa Gómez-Hernández, Cristina E. Rivas Duarte, José María Fernández García-Hierro, Marta G. Fuentes, Oscar Colmenares, Clara Forcada Barreda, Francisco Gómez Valle, Irene Jiménez García, Marcelo F. Jiménez

**Affiliations:** ^1^ Service of Thoracic Surgery, Salamanca University Hospital, Salamanca, Spain; ^2^ Salamanca Institute of Biomedical Research, Salamanca, Spain; ^3^ Department of Surgery, University of Salamanca, Salamanca, Spain; ^4^ Service of Radiology, Salamanca University Hospital, Salamanca, Spain

**Keywords:** pulmonary marking, minimally invasive surgery, hybrid operating room, electromagnetic navigation bronchoscopy (ENB), cone beam computed tomography, percutaneous marking

## Abstract

**Background:**

Intraoperative identification of subsolid or small pulmonary nodules during minimally invasive procedures is challenging. Recent localization techniques show varying success and complications. Hybrid operating rooms (HORs), equipped with radiological tools, facilitate intraoperative imaging. This study compares the accuracy and safety of marking pulmonary nodules using electromagnetic navigation bronchoscopy (ENB) combined with Cone Beam Computed Tomography (CBCT) vs. CBCT-guided percutaneous marking (PM).

**Methods:**

This retrospective cohort study included patients with pulmonary nodules scheduled for minimally invasive resection in a HOR. Marking techniques included ENB assisted by CBCT and PM guided by CBCT. The study compared the success rate, procedure time, intraoperative complications and radiation dose of both techniques.

**Results:**

A total of 104 patients with 114 nodules were included (October 2021—July 2024). Thirty nodules were marked using ENB, and 84 with PM. One case used both techniques due to ENB failure. No differences among groups were found in nodule characteristics. Success rates were similar (93.3% in ENB group vs. 91.7% in PM group, *p* = 1). Marking took significantly longer time in the ENB group (median 40 min) compared to PM group (25 min, *p* = 0.007). Five (6%) patients in the PM group experienced intraoperative complications compared to none in the ENB (*p* = 0.323). Radiation dose was significantly higher in the ENB group (*p* = 0.002).

**Conclusions:**

ENB assisted by CBCT is a safe and effective technique, with success rates comparable to CBCT-guided PM, though it may result in longer procedural times and higher radiation doses.

## Introduction

The number of patients undergoing chest computed tomography (CT) scans has significantly increased over the past decade. As a result, the detection of incidental pulmonary nodules has risen steadily (1, 2). Although most detected nodules are benign and do not require further evaluation (3), some nodules necessitate surgical resection due to their potential for malignancy.

Furthermore, with the widespread implementation of CT lung cancer screening programs, the detection of potential cancers presenting as ground-glass nodules or small-sized pulmonary nodules is expected to rise. Conversely, surgical removal of the tumour remains the standard of care for patients with early-stage non-small cell lung cancer (NSCLC) (4), with minimally invasive techniques such as video-assisted thoracoscopic surgery (VATS) and robotic surgery now considered gold standard approaches (4, 5). In this context, sublobar resections have also been demonstrated to be effective for treating peripheral small-sized lesions (6, 7).

Nevertheless, the intraoperative localization of subsolid or small-sized pulmonary nodules remains a challenge during minimally invasive approaches, as these nodules are not visible on the visceral pleura and are difficult to palpate in deflated lungs. This situation has driven the need for intraoperative identification methods (8–10) when a nodule is deeply located or not palpable.

Therefore, many techniques have been developed to assist the localization of small pulmonary nodules and ground-glass opacities, using either percutaneous or endoscopic approaches (11, 12). CT-guided percutaneous localization procedures have traditionally been the primary method for localizing lung nodules, while electromagnetic navigation bronchoscopy (ENB) represents a newer endoscopic approach facilitating access and marking peripheral pulmonary lesions. However, these localization techniques often encounter issues like marker displacement or diffusion, which can reduce their success rates, and they are not free from complications such as pneumothorax. In contrast, the increasing utilization of hybrid operating rooms (HORs), where both surgical resection and localization procedures can be performed in a single setting, has shown higher success rates and lower complication risks due to the reduced interval between localization techniques and resection (13). Nevertheless, there is currently no consensus on a standard preoperative localization technique, and thoracic surgeons typically select a method based on factors such as technical complexity, and the availability of resources. Therefore, the aim of this study is to compare the accuracy and safety of marking pulmonary nodules using ENB combined with Cone Beam Computed Tomography (CBCT) vs. percutaneous marking (PM) guided by CBCT in a HOR.

## Materials and methods

### Ethical statement

The need for Clinical Research Ethics Committee approval for this project was waived according to our institutional regulations because the study was a retrospective cohort based on anonymized patient data.

### Study design, data source and patients

We conducted a single-centre observational, retrospective, and comparative cohort study. All data were obtained from an institutional prospective database. Quality control of the data was assured by two successive audits made by the quality control manager of the unit. The database included standardized definitions (14) for clinical and pathological variables, type of resection, as well as intraoperative and postoperative data.

The inclusion criteria consisted of patients aged ≥ 18 years old scheduled for minimally invasive pulmonary resection for any diagnosis between October 2021 and June 2024 in our centre, and who required an intraoperative marking in the HOR. The indications for marking were as follows: (1) subsolid nodules; (2) partly solid nodules; (3) solid nodules smaller than 10 mm and (4) nodules seated more than 20 mm deep from the visceral pleura, independently of size. Each nodule was localized with one or multiple markings (dye and hook/coil).

### Marking techniques

A multidisciplinary team of thoracic surgeons and radiologists discussed and determined the choice of marking technique for each patient, focusing primarily on factors such as the nodule's location, its proximity to the pleura, and patient characteristics. As a general rule, patients with emphysematous lung disease and nodules situated near blood vessels or in areas not easily accessible by the percutaneous approach (such as the costophrenic angle or beneath the scapula) underwent ENB marking. Conversely, nodules situated more than 20 mm deep from the visceral pleura were typically marked percutaneously.

**Figure 1:**
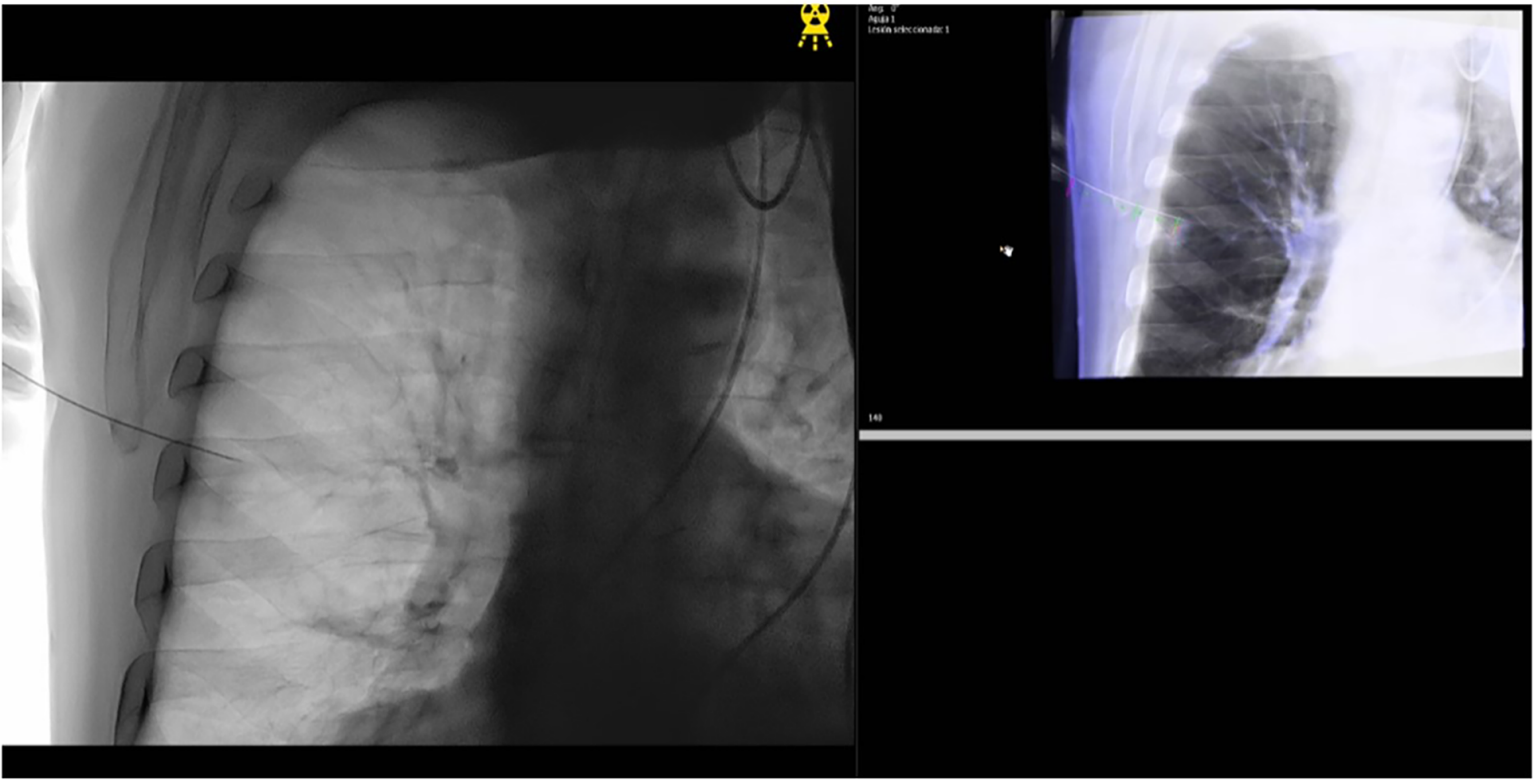
Imaging of electromagnetic navigation bronchoscopy (ENB) marking with cone beam computed tomography (CBCT) assistance.

**Figure 2:**
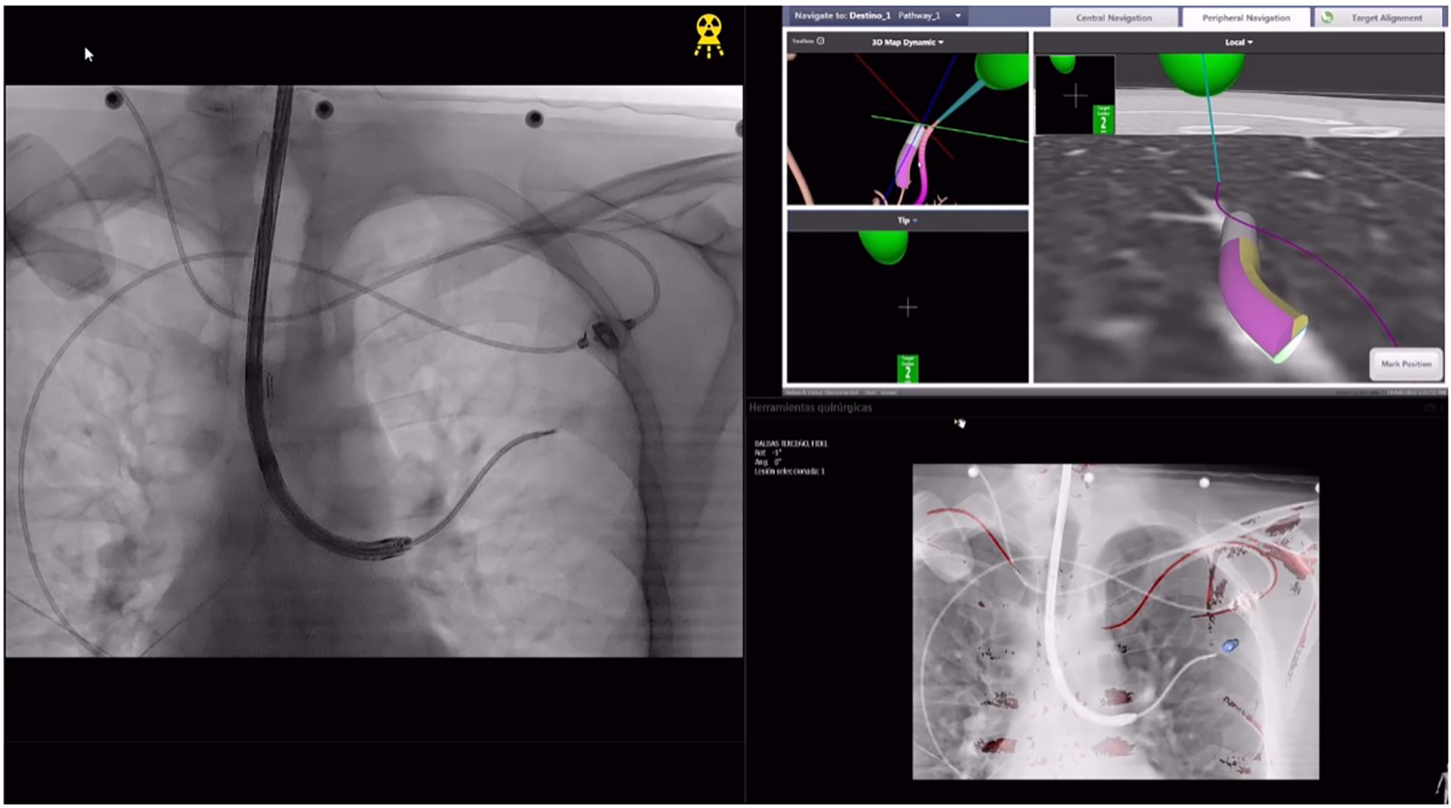
Intraoperative view of indocyanine green (ICG) marking performed with electromagnetic navigation bronchoscopy (ENB) and cone beam computed tomography (CBCT) assistance. This image is linked to Figure 1 and depicts the same case.

**Figure 3:**
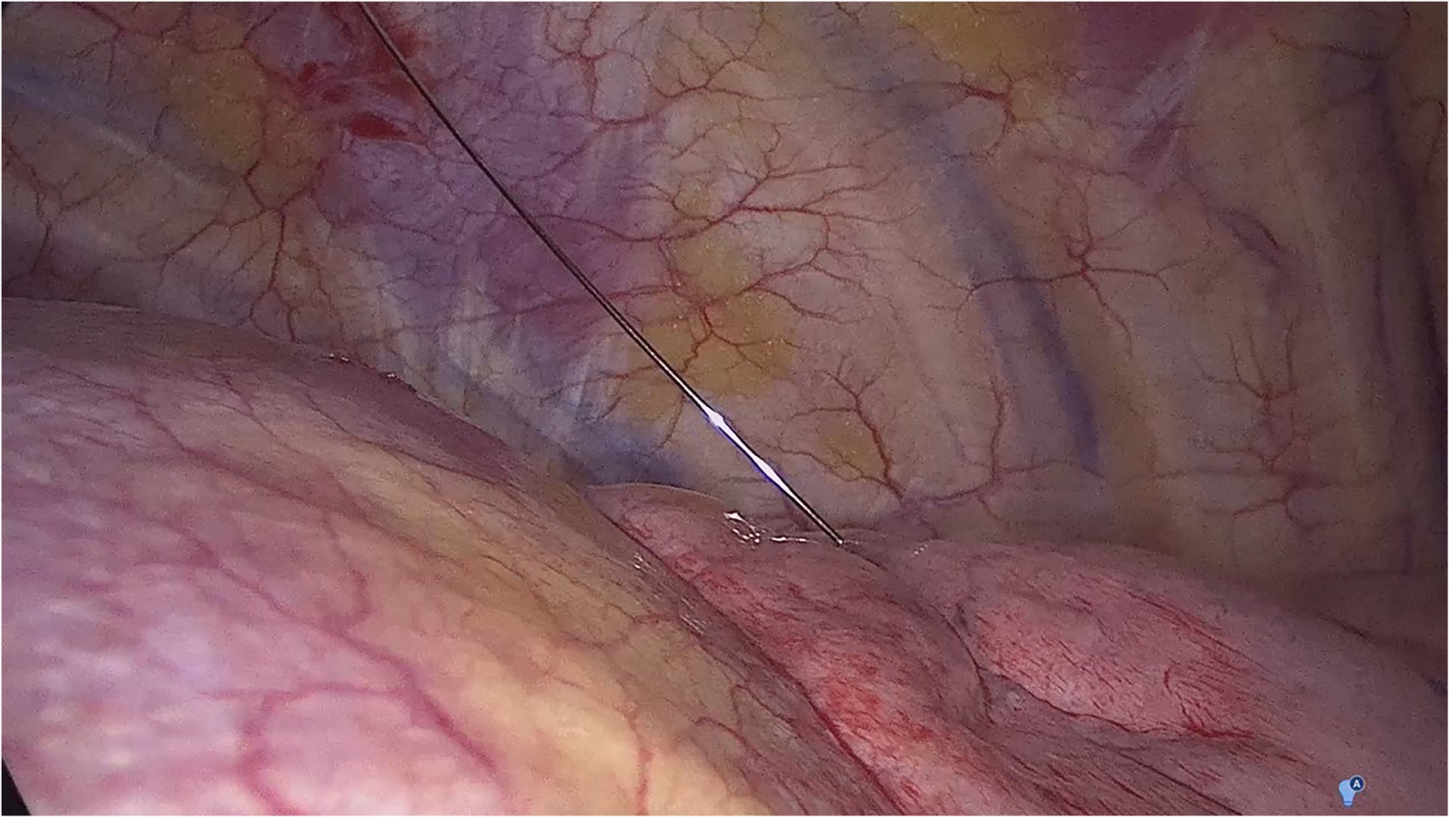
Imaging of percutaneous nodule marking guided by cone beam computed tomography (CBCT).

**Figure 4:**
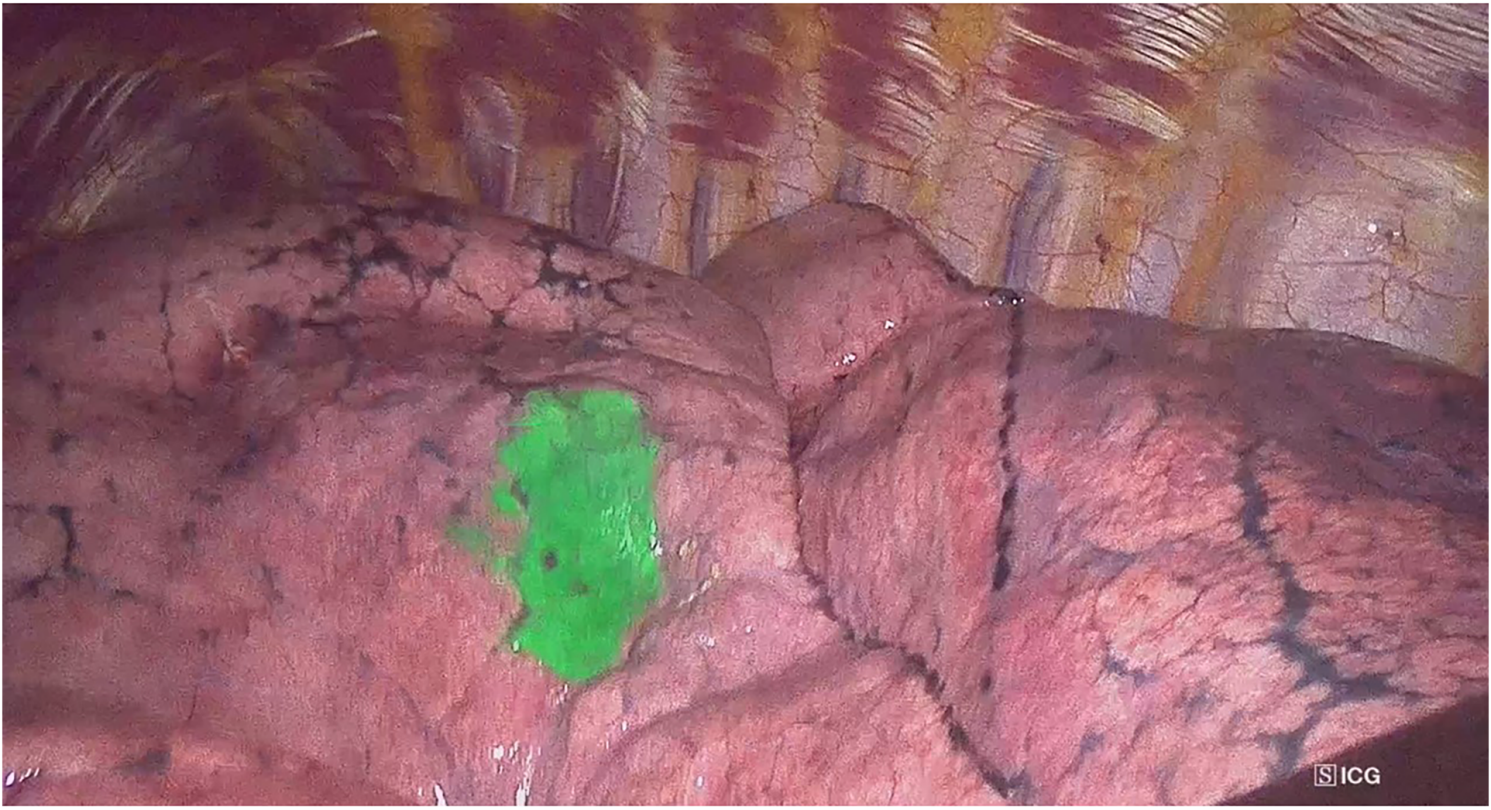
Intraoperative view of a hookwire after percutaneous marking guided by cone beam computed tomography (CBCT).

(a) ENB combined with CBCT (Figure 1): The ENB-guided dye marking was performed by one of the team surgeons (MFJ or MGF) in the HOR at the time of resection. A recent chest CT scan is required for planning, with a recommended acquisition protocol featuring a slice thickness of 1.0–1.25 mm and a slice interval of 0.8–1.0 mm. Axial, coronal, and sagittal views from the patient’s preoperative CT scan were utilized to plan the marking procedure. Generally, lesions within 1 cm of the pleural surface were marked directly. For lesions located more than 1 cm from the pleural surface, dye marking was performed midway between the lesion and the nearest pleural surface by creating a spherical virtual target at that point. When marking multiple nodules, a separate navigation pathway was created for each marking point. During the initial years of the study period, we used the superDimension system (Medtronic, Minneapolis, MN, USA), transitioning to the Illumisite platform (Medtronic, Minneapolis, MN, USA) from 2023 to 2024. The patient was positioned in the supine decubitus position, and general anaesthesia was induced with a laryngeal mask in the HOR. An intraoperative CT scan spin was performed using the CBCT (Azurion 7, Philips, Amsterdam, Netherlands), and segmentation of the target point was carried out with the XperCT reconstruction software. Subsequently, ENB was performed using the platform's software. Once the target was localized during navigation, augmented fluoroscopy was utilized to ensure the correct position of the locatable guide. The CBCT provided a real-time integration of conventional fluoroscopy image and the segmentation of the target point. Once the target lesion was accurately located using the combined ENB and CBCT, a new intraoperative CT scan spin was performed to confirm the precise position of the tip of the locatable guide. If no positional corrections were needed, the locatable guide was removed from the extended working channel under fluoroscopic guidance. A vial containing 25 mg of indocyanine green (ICG) was reconstituted with 20 ml of 20% human albumin, and a 1 ml syringe was preloaded with the dilution. A 21-gauge Arcpoint™ pulmonary needle (Medtronic, Minneapolis, MN, USA) was used for the injection. Approximately 0.5 ml of the ICG dilution was used to prime the needle path. The Arcpoint™ needle was then inserted into the extended working channel, and under fluoroscopic guidance, approximately 0.2 ml of the ICG mixture was injected into the target point using the preloaded syringe. After the lesion was localized and injected with ICG, the laryngeal mask was replaced with a double-lumen endotracheal tube. The patient was then positioned appropriately for minimally invasive resection (VATS or robotic surgery). Upon entering the chest, inspection of the lung was performed using either an endoscopic system equipped with an infrared fluorescence camera (Stryker, Kalamazoo, MI, USA) or the Firefly tool of the Da Vinci X platform (Intuitive Surgical, Sunnyvale, CA, USA) to visualize the ICG (Figure 2).
(b) PM guided by CBCT (Figure 3): The PM was performed by a radiologist specialised in chest pathology (JMF or IJ) in the HOR at the time of resection. The patient was appropriately positioned for minimally invasive resection and general endotracheal anaesthesia was induced using a doble-lumen tube. An intraoperative CT scan spin was performed using the CBCT (Azurion 7, Philips, Amsterdam, Netherlands) and marking planification of the target point was carried out with the XperGuide software tool. As a general rule, lesions within 1 cm of the pleural surface were marked directly with ICG. For lesions located more than 2 cm from the pleural surface, marking was performed using a hookwire or a coil. For marking planning, the target point was established at the level of the lesion, and the entry point was defined on the patient's skin surface. For direct dye marking, a 22-gauge × 100 mm needle (ChibaSono, Pajunk®, Geisingen, Germany) was used, while deeper lesions were marked using a 20-gauge × 107 mm needle with a 20 cm breast lesion localization hookwire (Curaway™, Zhejiang, China) or a Müller-Schimple Breast Localization Coil (19.5 gauge × 90 mm). During the PM procedure, the lung was kept inflated but not ventilated and the needle was inserted and advanced into the area adjacent to the target lesion under augmented fluoroscopic guidance. The CBCT was subsequently rotated using the XperGuide software to display the progression view, integrating conventional fluoroscopy images with the marking plan. Upon reaching the target lesion, either 0.15 ml of ICG diluted with 20% albumin to a concentration of 1.25 mg/ml was injected, or a hookwire or coil was inserted. In the latter case, a new intraoperative CT scan spin was performed to verify its correct positioning. Upon inspection of the chest using an endoscopic camera, the wire was visualized (Figure 4) or with the use of infrared fluorescence, the ICG was also identified. When using a hookwire or a coil, the resection of the lesion was guided not only by direct visualization but also by fluoroscopy assessment. To do this, we used a Forrester clamp to grasp the lung deeper than the wire tip or the coil, as a marker of our planned resection line, and used fluoroscopy to confirm that the coil or hook were well within the planned resection line, and stapled below this area. We found this to be essential for achieving an adequate margin.

In both approaches, the lesion was then resected with endoscopic staplers and the biopsy specimen was then evaluated by opening the specimen in the HOR and with pathologic confirmation by frozen-section analysis.

### Outcomes

The primary endpoint was success rate which was defined as the percentage of patients with successful identification of target lesions during the minimally invasive procedure. Secondary endpoints included conversion rate, procedural complications, time of the marking technique and radiation dose measured by the total radiation dose (mGy) and the dose area product (Gy. cm^2^).

### Statistical analysis

Demographic characteristics of patients and clinicopathological data and perioperative outcomes in each group were analysed and compared. Categorical data were summarized as frequency counts and percentages. All continuous variables were examined for normality using the Shapiro Wilk test and are shown as mean (SD: standard deviation) when normally distributed, and nonparametric data are presented as median (Q1–Q3). The frequencies of categorical variables were compared using the Chi-squared test or the Fisher's exact test, whereas continuous variables between groups were compared using Student's *t*-test when normally distributed or the Mann Whitney *U*-test otherwise. Statistical significance was set at *p* < 0.05. SPSS v28.0 (SPSS Inc., Chicago, IL, USA) was used for data analysis.

Our manuscript is reported according to the STROBE recommendations.

## Results

A total of 104 patients with 114 pulmonary nodules requiring intraoperative marking and operated on between October 2021 and July 2024 were included. Nine patients required multiple markings. All procedures consisted of sublobar resections. Table 1 shows demographic and clinical characteristics of the patients, while Table 2 details nodule characteristics.

**Table 1:** Clinical characteristics of the study population.

	ENB + CBCT *N* = 28	PM + CBCT *N* = 76	*P*-value
Age, median (IQR)	70.5 (59–78.5)	69 (65–74)	0.947*
Male sex, *n* (%)	21 (75)	46 (60.5)	0.171**
BMI, median (IQR)	23.95 (22.2–27.97)	25.47 (22.84–28.21)	0.595*
FEV1%, median (IQR)	95.5 (73.78–105.25)	93 (73–108)	0.98*
DLCO%, median (IQR)	83.5 (69–92.75)	88 (71.5–103.5)	0.327*
Previous ipsilateral surgery, *n* (%)	1 (3.6)	8 (10.5)	0.439***

ENB, electromagnetic navigation bronchoscopy; CBCT, cone beam computed tomography; PM, percutaneous marking; BMI, body mass index; FEV1, forced expiratory volume in the first second; DLCO, diffusing capacity of the lungs for carbon monoxide; IQR, interquartile range.

* *p*-value for Mann-Whitney *U*-test.

** *p*-value for Chi-squared test.

*** *p*-value for Fisheŕs exact test.

**Table 2:** Characteristic of the target nodules requiring marking.

	ENB + CBCT *N* = 30	PM + CBCT *N* = 84	*P*-value
CT characteristics, *n* (%):			0.25**
- GGO	4 (13.3)	22 (26.2)	
- Solid	23 (76.7)	58 (69)	
- Mixed	3 (10)	4 (4.8)	
Tumour size, mm, median (IQR)	9.5 (5.75–12.75)	8.75 (7–10)	0.522*
Depth from visceral pleura, mm, median (IQR)	7 (2.75–12)	6 (2–16.75)	0.882*
Tumour location, *n* (%)			0.027**
Upper/middle lobe	22 (73.3)	42 (50)	
Inferior lobe	8 (26.7)	42 (50)	
Type of resection, *n* (%)			0.758***
Wedge	27 (90)	73 (86.9)	
Segmentectomy	3 (10)	11 (13.1)	
Histology, *n* (%)			0.132**
Lung cancer	11 (36.7)	42 (50)	
Pulmonary metastasis	12 (40)	34 (40.5)	
Other	7 (23.3)	8 (9.5)	

ENB, electromagnetic navigation bronchoscopy; CBCT, cone beam computed tomography; PM, percutaneous marking; GGO, ground-glass opacity; IQR, interquartile range.

* *p*-value for Mann-Whitney *U*-test.

** *p*-value for Chi-squared test.

*** *p*-value for Fisheŕs exact test.

Thirty (26.9%) of the nodules were marked by ENB assisted by CBCT, and 84 (77.3%) with PM. In one case, a combination of both techniques was performed due to the failure of ENB marking (the target point could not be reached). PM was primarily performed using ICG in 46 cases (54.8%), while hookwires and coils were used in 32 cases (38.1%) and 6 cases (7.1%), respectively.

Table 3 shows outcomes of both marking procedures. No differences were detected between the ENB and the PM group in the proportion of subsolid lesions (13.3% vs. 26.2%, *p* = 0.25), nodule size (9.5 mm vs. 8.75 mm, *p* = 0.522), or the distance of the marked nodule to the visceral pleura (7 mm vs. 6 mm, *p* = 0.882). The majority of the nodules marked with ENB were located in the upper lobes (73.3%, *p* = 0.027). The duration of the marking technique was significantly longer in the ENB group with a median of 45 min compared to 25 min in the PM group (*p*=<0.001). Marking was successful in 28/30 (93.3%) nodules in the ENB group vs. 77/84 (91.7%) in the PM group (*p* = 1). One patient required conversion to open approach in the PM group due to intense pleural adhesions. Five (6%) patients in the PM group experienced intraoperative complications (pneumothorax, lung tear, and subclavian vein puncture after hook mobilization) compared to none in the ENB (*p* = 0.323). Radiation dose and dose product area (DPA) were significantly higher in the ENB (*p* = 0.002 and *p* = 0.002, respectively).

**Table 3:** Outcomes of the two marking techniques.

	EBN + CBCT *N* = 30	PM + CBCT *N* = 84	*P*-value
Success rate, *n* (%)	28 (93.3)	77 (91.7)	1***
Conversion, *n* (%)	0 (0)	1 (1.2)	1***
Complications, *n* (%)	0 (0)	5 (6)	0.323***
Pneumothorax		3	
Lung laceration		1	
Subclavian vein puncture		1	
Marking time, min, median (IRQ)	45 (35–88)	25 (17–40)	<0.001*
Radiation dose, mGy, median (IQR)	77.7 (54.2–181)	51.4 (23.4–106)	0.002*
DAP, Gy.cm^2^, median (IQR)	29.5 (19.9–69)	19.5 (8.51–40.4)	0.002*

ENB, electromagnetic navigation bronchoscopy; CBCT, cone beam computed tomography; PM, percutaneous marking; IQR, interquartile range; DAP, dose área product.

* *p*-value for Mann-Whitney *U*-test.

** *p*-value for Chi-squared test.

*** *p*-value for Fisheŕs exact test.

## Discussion

Localizing small, deep, or subsolid nodules during minimally invasive surgery poses significant challenges, as these lesions are often neither easily visible nor palpable, making accurate localization crucial for thoracic surgeons to perform sublobar resections with adequate margins and within a short procedural time. In this context, HORs have emerged as a valuable tool for thoracic surgeons, enhancing the success rates of localization techniques while minimizing morbidity. Additionally, conducting the marking procedure in a hybrid operating room (HOR) enhances patient comfort by utilizing general anaesthesia and facilitates a seamless transition between localization and surgery, eliminating the need to change settings and thereby reducing both procedure time and associated risks. In the current study, we compared two marking techniques commonly used in HORs and observed favourable success rates in both the PM and ENB marking groups, each exceeding a 90% success rate.

Our experiences and findings support the results of prior research documenting several techniques for lesion localization. These techniques have been primarily categorized into percutaneous and transbronchial approaches, using either dyes or metallic devices. Each method presents its own set of benefits and drawbacks. In the present work, the selection of the type of marking was agreed upon by a team of surgeons and radiologists and was based on the characteristics of the target nodule and the patient.

CT-guided PM is a well-established technique with a success rate of up to 90% and relatively low costs (15, 16). However, its major drawback is the risk of complications from pleural puncture, which complicates the localization of multiple lesions. Therefore, the PM approach was avoided in patients with emphysema and when the lesion was near a vessel due to the risk of air embolism or bleeding. Despite these precautions, three patients developed pneumothorax during the PM procedure, visible on fluoroscopy or CT. None of these cases compromised patient stability, as the lung was not ventilated and the surgery was promptly performed.

Therefore, ENB was used for patients with emphysematous lung disease and nodules located near a vessel since it is associated with less complications such as pneumothorax and bronchial haemorrhage (17). In our series, no complications were encountered with ENB marking. Additionally, the bronchoscopic approach was chosen to access regions challenging for the percutaneous method, such as nodules near the costophrenic angle, beneath the scapula, on the mediastinal side, and in the craniodorsal area obscured by the scapula (18). However, ENB was avoided for nodules in the lower lobes due to the difficulty in accessing them caused by CT-to-body divergence (19). Based on our experience and as previously reported (20–22), the ENB-guided technique has proven to be a feasible method for managing lung nodules and it can be consider as a viable alternative for the preoperative localization of small pulmonary nodules (15). Moreover, ENB allows localization of multiple lung nodules (>2 lung nodules) or bilateral lung lesions (20, 23). Conversely, it is less cost-effective, more labour-intensive, and demands a high level of skill and experience from the operator, even after mastering the learning curve. This complexity limits its availability in many institutions.

The selection of the marking tracer was also coordinated with both the surgical and radiological teams. Briefly, peripheral nodules (<10 mm from visceral pleura) were marked with dye, while deeper nodules were marked with a hookwire or a coil. We chose ICG as a dye since, in our previous experience, methylene blue, although effective, safe, and inexpensive, may easily diffuse away from the nodule staining the entire pleural cavity. In contrast, ICG is a fluorophore that absorbs and emits light at 820 nm, and it is detectable exclusively by a near-infrared (NIR) camera (24). Nevertheless, we encountered two cases of unsuccessful ICG marking. In one case, dye diffusion into the pleural cavity occurred due to misinjection. In another case, the marking was obscured by intense pleural adhesion between the lung and pleura. Diffusion into adjacent parenchyma was not observed in patients with emphysema or bullae, as the ICG was diluted in albumin to increase its concentration. In a subsequent case, localization was also obscured, likely due to dye injection far from the pleura. We resolved this by manipulating the surface with soft gauze based on anatomical location to reveal the dye marking.

For nodules located more than 10 mm deeper than the pleural surface, we used hookwires or coils, as hookwire needle localization has been demonstrated to be comparable to methylene blue injection in terms of success and complication rates (25). However, complications such as pneumothorax and pulmonary hemorrhage remain significant concerns. In our series, we encountered two cases of unsuccessful marking with this method. In one case, the hookwire was inadvertently dislodged from the lung parenchyma when it was released from the chest wall. This occurred because, if the distance between the wire tip and the pleura is less than 30 mm, the wire may fail to dock properly due to insufficient friction between the wire and the pulmonary tissues (26). Additionally, a serious complication occurred when the hookwire punctured the subclavian vein during specimen manipulation prior to extraction.

More recently, the use of microcoils for pulmonary nodule localization has been introduced as a painless and convenient option (26). Outside the context of HORs, microcoils can be placed days before surgery, facilitating preoperative scheduling and subsequent surgical resection. However, this method has some drawbacks, including risks of dislodgement, pneumothorax, intrapulmonary haemorrhage, and pleural pain.

In our series, we used microcoils along with other markers, such as ICG, to identify the area where the coil was positioned and utilized intraoperative fluoroscopy to ensure accurate nodule resection. However, we experienced two cases where the microcoils were unsuccessful due to dislodgement.

It is important to note that in one case, ENB marking was unsuccessful because the probe could not reach the target nodule. As a result, we decided to perform PM instead. The HOR provides the flexibility to switch to the most appropriate marking technique if the initial method failed and also allows for the combination of multiple marking techniques within the same surgical procedure.

Consequently, we do not suggest a single primary technique and recommend that decision-making be based on the individual patient's condition. Specifically, factors such as nodule's location, distance from the pleura, size, patient characteristics, CT findings and cost should be considered when choosing localization techniques.

Our study had several potential limitations. Firstly, the retrospective design imposes constraints and introduces selection bias. Secondly, the ENB-guided technique was used in only about 30% of patients. Thirdly, the success rate may have been influenced by the learning curve of the physicians responsible for the marking procedure. Additionally, data were obtained from a single public institution, and the sample size was too small to demonstrate a clinically significant difference, even though statistical significance was achieved. Further large-scale studies directly comparing different techniques are necessary to establish a standard for marking pulmonary nodules. Despite these limitations, our study reflects real-world experience and the decision-making process involved.

## Conclusion

In the current study, ENB marking combined with CBCT proved to be a safe and effective technique, with success rates comparable to PM guided by CBCT. While it is associated with a lower risk of complications, it may result in slightly longer surgical times and higher radiation doses. Therefore, we do not recommend a single primary technique and decision-making should depend on the individual patient's characteristics.

## Data Availability

The raw data supporting the conclusions of this article will be made available by the authors, without undue reservation.
